# Prognostic significance of soluble mesothelin in malignant pleural mesothelioma: a meta-analysis

**DOI:** 10.18632/oncotarget.17436

**Published:** 2017-04-26

**Authors:** Long Tian, Rujun Zeng, Xin Wang, Cheng Shen, Yutian Lai, Mingming Wang, Guowei Che

**Affiliations:** ^1^ Department of Thoracic Surgery, West China Hospital, Sichuan University, Chengdu, China; ^2^ Department of Obstetrics and Gynecology, West China Second University Hospital, Sichuan University, Chengdu, China

**Keywords:** soluble mesothelin, malignant pleural mesothelioma, prognosis, meta-analysis

## Abstract

**Background:**

Soluble mesothelin is beneficial to detect the progression and the treatment response of malignant pleural mesothelioma. However, the prognostic value of soluble mesothelin in malignant pleural mesothelioma remains unclear.

**Methods:**

Hazard ratio with 95% CI was used to evaluate the prognostic value of soluble mesothelin and the effect of clinicopathological characteristics on the survival of malignant pleural mesothelioma.

**Results:**

Eight eligible studies involving 579 patients were selected for this meta-analysis. The results showed that soluble mesothelin level was significantly correlated with the survival of malignant pleural mesothelioma (pooled HR: 1.958, 95%CI: 1.531-2.504, p = 0.000; heterogeneity test: I^2^ = 1.1%, p = 0.421). In addition, the survival of malignant pleural mesothelioma was significantly correlated with some clinicopathological characteristics such as tumor histology (HR = 3.214, 95% CI = 2.071-4.988, p = 0.000; heterogeneity test: I^2^ = 0.0%, p = 0.623) and tumor stage (HR = 2.007; 95% CI = 1.477-2.727; p = 0.000; heterogeneity test: I^2^ = 0.0%, p = 0.966).

**Conclusions:**

The survival of malignant pleural mesothelioma is significantly correlated with tumor histology and tumor stage. Furthermore, high soluble mesothelin level may lead to a poor prognosis for malignant pleural mesothelioma patients.

## INTRODUCTION

Malignant pleural mesothelioma(MPM) is a rare but highly aggressive asbestos-induced malignancy, with a poor prognosis and increasing incidence [1]. Although the surgery and combination chemotherapy are proved effective, the median overall survival of MPM remains poor [2]. Therefore, it is necessary to identify prognostic markers to help with the survival of MPM. Mesothelin is a 40 kDa cell surface glycoprotein, and highly expressed in several human cancers, including mesotheliomas, pancreatic cancers and ovarian cancers [3]. Although mesothelin bounds to cell membrane, a circulating form termed soluble mesothelin can be detected in the blood and pleural effusion by using enzyme-linked immunosorbent assay (ELISA) [4]. Soluble mesothelin is a useful biomarker, which can not only play an important role in the diagnosis [5, 6], but also be beneficial to monitor the progression and treatment response of MPM [7]. Recently, some studies have reported that high soluble mesothelin level can be considered as a negative prognostic factor in patients with MPM [8–12]. On the other hand, some studies show that the prognostic value of soluble mesothelin in MPM is not conclusive and needs further evaluation [13–15]. Despite numerous published studies, the prognostic value of soluble mesothelin remains controversial. Additionally, the correlation between the survival of MPM patients and clinicpathological characteristics such as age, gender, tumor histology and tumor stage are uncertain. To evaluate the prognostic value of soluble mesothelin and the effect of clinicopathological characteristics on the survival of MPM patients, we performed a meta-analysis.

## RESULTS

### Characteristics of eligible studies

**Figure 1:**
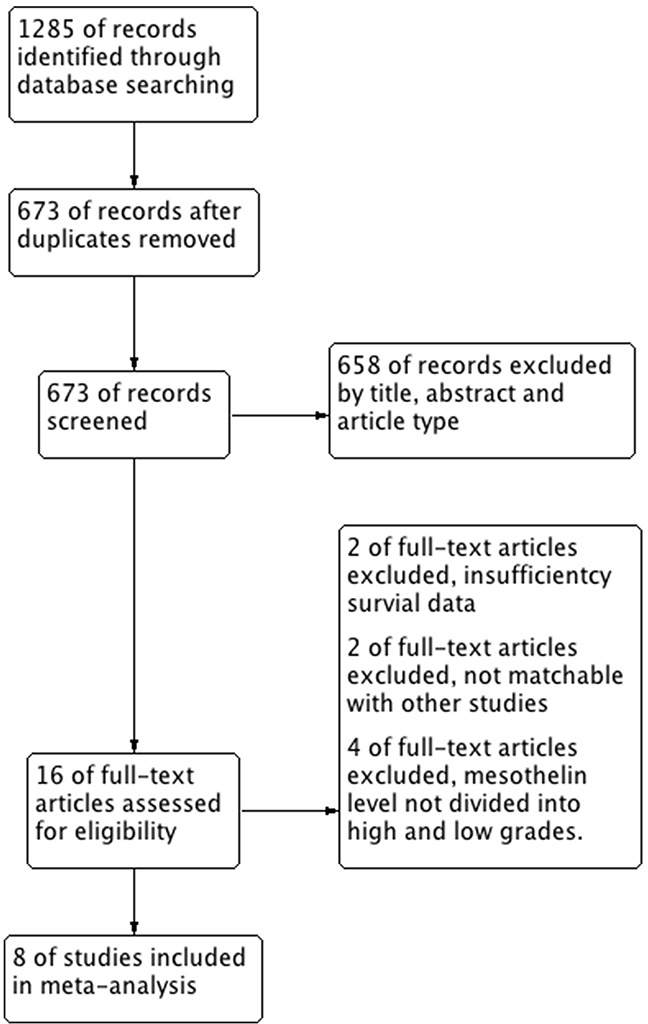
Flow chart of study selection in this meta-analysis

A total of 1285 records were identified through database searching. Six hundred and twelve records were excluded for duplication. After browsing the titles and abstracts, 16 studies that might meet the inclusion criteria were screened out to read full texts. These studies were carefully reviewed. Finally, 8 eligible studies including 579 patients were selected for this meta-analysis (Figure 1). The characteristics of eligible studies were summarized in Table 1. The size of sample in each study ranged from 36 to 107 and all of the patients were Caucasian. The most common tumor site of the patients was pleural. The mestothelin level was tested using serum specimen in 7 studies. In the rest 1 study, the samples were from pleural effusion. Two Mesomark ELISA kits (Mesomark kit by Fujirebio Diagnostics, Malvern, Pennsylvania, USA or by Cisbio International, Gif-Sur-Yvette, France) were used in eligible studies to measure mesothelin level. The mesothelin level was expressed in nanomoles per liter (nmol/L) in the eligible studies. Six studies provided available HR with 95%CI. The other 2 studies had their HRs and 95%CIs calculated from Kaplan- Meier survival curves.

**Table 1:** Main characteristics and results of the eligible studies

Author (year)	Country	Studytype	No. ofpatients	Ethnicity	TumorSite	Tumorhistology(Epi/Sar/Bip/others)	Tumorstage (I/II/III/IV)	Treatment	Specimen	Cut-off(nmol/L)	Method	Sourceof HR	Multivariateanalysis	Qualityscore
Linch et al.(2014) [14]	UK	P	53	Caucasian	**Ple/Per**49/4	46/1/5/1	NA	CT/BSC30/23	Serum	2.7	ELISA^a^	Reported	No	7
Dipalma et al. (2011) [11]	Italy	P	36	Caucasian	pleural	29/4/3/0	16/6/3/11	NA	Serum	1.2	ELISA^b^	Reported	No	7
Creaney et al. (2011) [10]	Australia	P	95	Caucasian	pleural	68/9/18/0	NA	CT/BSC /Surg/RT61/25/7/2	Serum	5	ELISA^a^	Reported	Yes	9
Grigoriu et al. (2009) [29]	FranceUSA	R	40	Caucasian	pleural	35/3/2/0	NA	CT/BSC/GT19/5/16	Serum	NA	ELISA^c^	Estimated	No	6
Schneider et al. 2008) [12]	Germany	P	100	Caucasian	pleural	66/12/15/7	I/II/III/IV/II-III6/20/36/14/24	CT/Surg/RT/BSC/Un68/14/2/9/7	Serum	3.5	ELISA^a^	Reported	Yes	8
Grigoriu et al. (2007) [9]	France	P	96	Caucasian	pleural	73/10/13/0	11/21/32/19	CT/BSC /Surg70/16/10	Serum	3.5	ELISA^b^	Reported	Yes	8
Cristaudo et al. (2007) [8]	Italian	P	107	Caucasian	pleural	72/10/7/18	I-II/III-IV/NOS* 43/45/19	NA	Serum	1	ELISA^b^	Reported	Yes	9
Creaney et al. (2007) [13]	Australia	P	52	Caucasian	NA	15/9/5/23	NA	NA	PE	26	ELISA^a^	Estimated	No	7

P: prospective; R: retrospective; Ple: pleural; Per: peritoneal; Epi: epithelial; Sar: Sarcomatoid; Bip: Biphasic; NA: not available; Un: unknown; NOS*: not otherwise specified; PE: pleural effusion; CT: chemotherapy; RT: radiotherapy; BSC: best supportive care; Surg: surgery; GT: immunotherapy.

ELISA^a^: tested by the MESOMARK kit (Fujirebio Diagnostics); ELISA^b^: tested by the MESOMARK kit (Cisbio International); ELISA^c^: tested by the MESOMARK kit (Fujirebio Diagnostics or Cisbio International).

### Prognostic impact of soluble mesothelin in MPM

**Figure 2:**
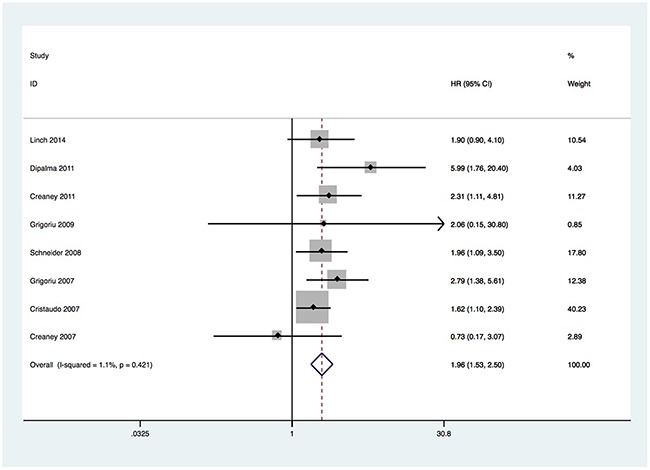
Forest plots for the correlation between soluble mesothelin and overall survival

The main meta-analysis results, the correlation between soluble mesothelin and the survival of MPM, were summarized in Figure 2. The pooled HR for all eligible studies which evaluated soluble mesothelin in MPM patients was 1.958 (95%CI 1.531-2.504). The results showed that high soluble mesothelin level was statistically significant correlated with the overall survival of MPM (p = 0.000) with a low heterogeneity (I^2^ = 1.1%, p = 0.421). To detect the potential heterogeneity, subgroup analysis stratified by specimen type, cut-off value, sample size, source of HR and survival analysis mode were applied. The results of subgroup analysis indicated that patients with high soluble mesothelin level had poorer overall survival compared with patients with low soluble mesothelin level in the following subgroups: serum subgroup (HR = 2.016; 95% CI =1.571-2.588; p = 0.000), cut-off value <5 subgroup (HR = 1.980; 95% CI = 1.516-2.586; p = 0.000), sample size <50 subgroup (HR = 4.970; 95% CI = 1.633- 15.131; p = 0.005), sample size ≥50 subgroup (HR = 1.866; 95% CI = 1.450- 2.402; p = 0.000), HR reported subgroup (HR = 2.016; 95% CI = 1.569-2.591; p = 0.000), multivariate analysis subgroup (HR = 1.925; 95% CI = 1.466-2.528; p = 0.000), non-multivariate analysis subgroup (HR = 2.111; 95% CI = 1.188-3.752; p = 0.011). The results of subgroup analysis revealed some possible contributors to the heterogeneity: sample size, specimen type, source of HR and survival analysis mode. The detailed results of subgroup analysis were summarized in Table 2.

**Table 2:** Pooled hazard ratio (HR) of soluble mesothelin (high vs low level) for overall survival according to subgroup analysis

Subgroup	No. of studies	Effects model	HR(95%CI)	Significance	Heterogeneity test		
					Chi^2^	I^2^ (%)	p-Value
Overall	8	Fixed	1.958 (1.531, 2.504)	p = 0.000	7.08	1.1	0.421
Specimen							
Serum	7	Fixed	2.016 (1.571, 2.588)	p = 0.000	5.24	0.0	0.514
Pleural effusion	1	-	0.73 (0.17, 3.07)	-	-	-	-
Cut-off value (nmol/L)							
<5	5	Fixed	1.980 (1.516, 2.586)	p = 0.000	5.09	21.4	0.278
≥5	2	Fixed	1.826 (0.949, 3.511)	p = 0.071	1.94	48.4	0.164
NA	1	-	2.02 (0.14, 29.97)	-	-	-	-
Sample size							
<50	2	Fixed	4.970 (1.633, 15.131)	p = 0.005	0.51	0.0	0.475
≥50	6	Fixed	1.866 (1.450, 2.402)	p = 0.000	3.74	0.0	0.587
Source of HR							
Reported	6	Fixed	2.016 (1.569, 2.591)	p = 0.000	5.24	4.5	0.388
Estimated	2	Fixed	0.925 (0.259, 3.297)	p = 0.904	0.45	0.0	0.502
Multivariate analysis							
Yes	4	Fixed	1.925 (1.466, 2.528)	p = 0.000	2.07	0.0	0.558
No	4	Fixed	2.111 (1.188, 3.752)	p = 0.011	4.93	39.1	0.177

### Correlation between clinicopathological characteristics and the survival of MPM

The main meta-analysis results of the correlation between clinicopathological characteristics and the survival of MPM patients were summarized in Table 3. The clinicopathological characteristics included age, gender, tumor histology and tumor stage. The results showed that not all the clinicopathologicl characteristics were significantly correlated with the survival of MPM patients. The pooled HR for age, gender, tumor histology and tumor stage were, respectively, 1.256(95% CI =0.907-1.739; p = 0.170), 0.932(95% CI =0.168-5.17; p = 0.936), 3.214(95% CI = 2.071-4.988; p = 0.000), 2.007(95% CI = 1.477-2.727; p = 0.000). No significant correlations were found between the survival of MPM patients and age or gender. However, tumor histology and tumor stage were significantly related to the survival of MPM patients with no heterogeneity.

**Table 3:** Main results of correlation between clinicopathological characteristics and the survival of MPM patients

Clinical characteristics	No. of studies	Effects model	Pooled HR(95%CI)	Significance	Heterogeneity test		
					Chi^2^	I^2^ (%)	p-Value
Age(<65 years vs >65 years)	3 [8] [10] [12]	Random	1.256 (0.907, 1.739)	p = 0.170	6.76	70.4	0.034
Gender(Male vs female)	2 [10] [12]	Random	0.932 (0.168, 5.17)	p = 0.936	9.04	88.9	0.003
Tumor histology(Epithelioid vs non-epithelioid)	3 [9] [10] [12]	Fixed	3.214 (2.071, 4.988)	p = 0.000	0.95	0.0	0.623
Tumor stage(I-II vs III-IV)	3 [8] [10] [12]	Fixed	2.007 (1.477, 2.727)	p = 0.000	0.07	0.0	0.966

### Sensitivity analysis and publication bias

**Figure 3:**
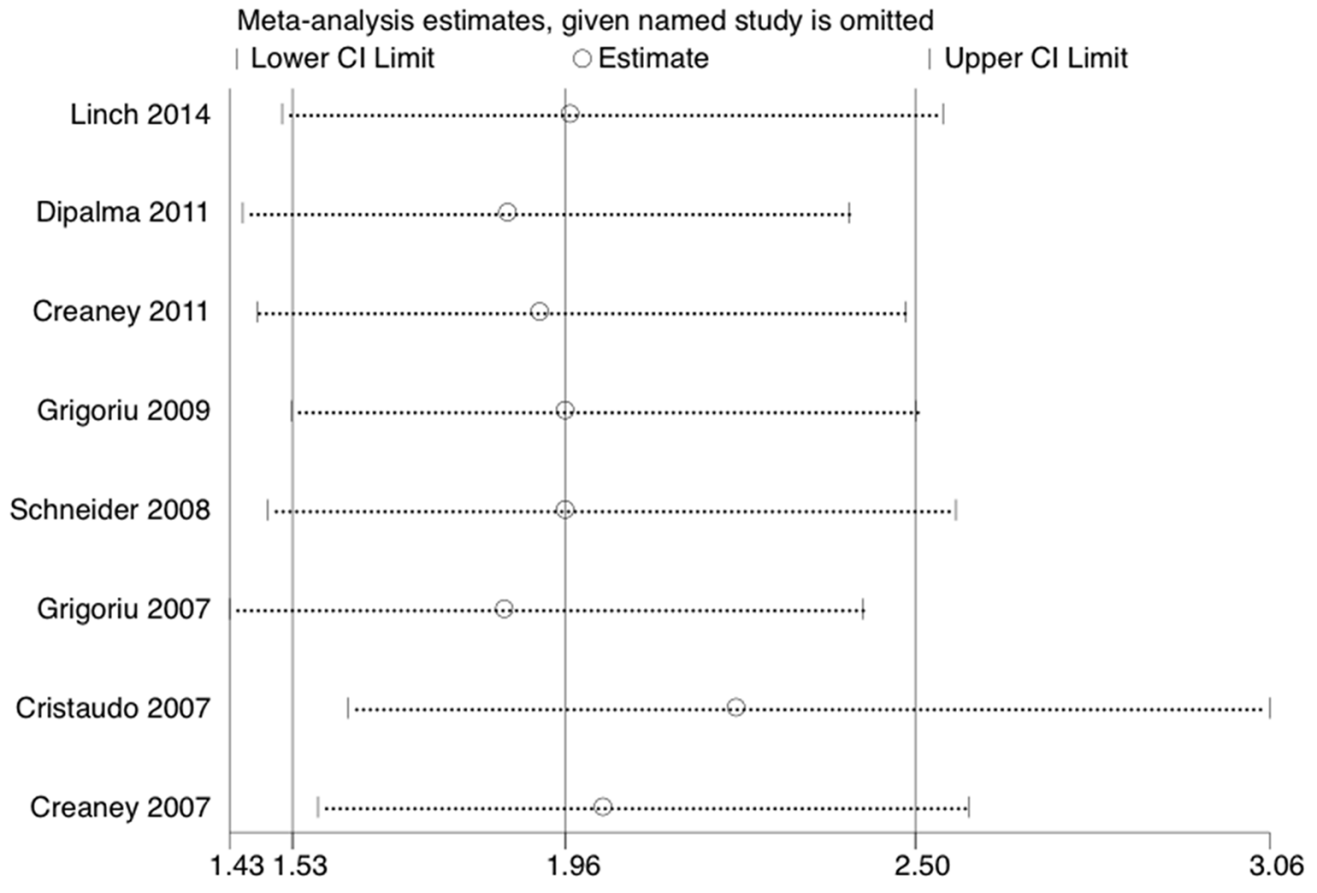
Sensitivity analysis on the correlation between soluble mesothelin and overall survival

**Figure 4:**
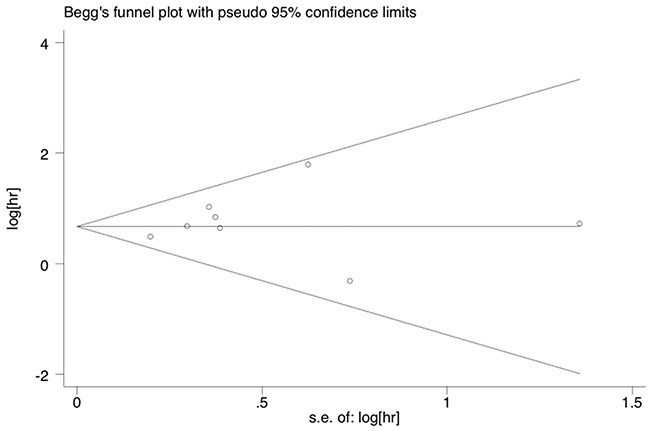
Funnel plots of publication bias on the correlation between soluble mesothelin and overall survival

Sensitivity analysis was used to estimate the influence by omitting one study at a time and calculate the combined HR. The result indicated that no individual study had a significant influence on the observed effect size (pooled HR) for overall survival (Figure 3). Begg's funnel plot with pseudo 95 % confidence limits was performed to estimate the publication bias of the eight studies included about overall survival (Figure 4). There was no significant publication bias observed with the Begg's tests of overall survival (p = 0.711). To validate the result, the nonparametric trim and fill method was further used to evaluate the impact of this bias and the pooled HR for overall survival. The results indicated no significant publication bias among studies and the pooled HR remained significant. Collectively, the result of this meta-analysis was robust and statistically reliable.

## DISCUSSION

To our best knowledge, this meta-analysis is the first study to evaluate the prognostic value of soluble mesothelin and effect of clinicopathological characteristics on the survival of MPM patients.

Mesothelin was firstly studied as a prognostic indicator in MPM by Cristaudo et al. [8], who reported a significant inverse correlation between mesothelin level and the overall survival of MPM. Grigoriu et al. [9] also found high mesothelin level was negatively correlated with the survival of MPM patients in both univariate and multivariate analysis. Another study, conducted by Yamada et al. [16], indicated that pleural effusion mesothelin level also had a prognostic effect the survival of MPM patients. However, in two studies conducted by Hollevoet et al. [17, 18], finding serum mesothelin was not an effective predictor for the survival of MPM patients. By the result of our meta-analysis, we found that high mesothelin level is a negative prognostic factor for MPM patients. Moreover, in the subgroup analysis, which survival analysis was conducted by multivariate analysis, the high mesothelin level was still significantly related to the survival of MPM patients. Data above indicated that mesothelin might be an independent prognostic factor in MPM patients. In addition, some clinicopathdological characteristics such as age, gender, tumor histology and tumor stage were also reported to have prognostic effects on the survival of MPM Patients. Recent studies showed old age, male gender, non-epithelioid histology and advanced tumor stage were significantly related to poor survival of MPM patients [19–22]. The results of our meta-analysis indicated that the survival of MPM patients was significantly correlated with tumor histology and tumor stage, which suggested that non-epithelioid histology and advanced tumor stage were significant negative predictors of the survival in MPM patients. However, neither age nor gender was found to be significantly related with the survival of MPM patients in our meta-analysis. There are some other independent prognostic factors proposed for MPM patients, such as white blood cell count, C-reactive protein level, hemoglobin level, neutrophil-to-lymphocyte ratio, lactate dehydrogenase, performance status, comorbidity, weight loss and breathlessness [20, 23–26]. Moreover, the therapies also contributed to the survival of MPM patients. Patients treated with chemotherapy had significantly longer survival than those treated with best supportive care [26]. Chemotherapy and radiation to port sites independently and in combination were associated with improved overall survival in MPM patients [27]. Absence of adjuvant therapy and non-curative surgery were significantly related to poor survival [19].

The mechanisms responsible for the association between high mesothelin level and poor survival of MPM patients remain unclear. More and more studies have showed that soluble mesothein level was closely associated with disease progression, disease status and tumor burden [17, 28, 29]. Additionally, Mesothelin contributes to further understanding of the biology of MPM, because it participates in cell adherence, cell proliferation and tumor invasion of MPM [30, 31]. Mesothelin is a suitable candidate for drug therapy and a potential target for designing novel therapeutic strategies [32]. Recent studies have indicated that antimesothelin immunotoxins, such as SS1P and RG7787, are well tolerated and exhibits significant antitumor activity in patients with MPM [33, 34]. Amatuximab is tolerable in MPM patients with a disease control rate of 90% when given with pemetrexed and cisplatin [35, 36]. The exposure of amatuximab is associated with overall survival, higher amatuximab exposure accompanied with longer overall survival [37]. Mesothelin gene silencing has an antitumor effect on cell lines overexpressing mesothelin deriving from MPM, which means mesothelin could be considered as a key molecular target for novel gene-based targeted therapies of MPM [38]. Collectively, mesothelin participates in cell adherence, cell proliferation, tumor invasion of MPM, and plays an important role in monitoring the treatment response, the disease progression and the prognosis in MPM patients. Mesothelin is also a suitable potential target for drug therapy.

There are some limitations in this meta-analysis. Firstly, both the number of included studies and the simple size of each study were relatively small. Secondly, not all of the HRs with 95% CI were directly extracted from the studies, the reconstructed HR calculated via Kaplan-Meier survival curves unavoidably brought some unexpected errors. Thirdly, the ethnicities of the patients in our analysis were Caucasian, there might be ethnic bias existed. Fourthly, not all of the survival analyses of the eligible studies were conducted by the multivariate analysis, there might be some confounding factors existed. Fifth, though no significant heterogeneity among studies was found in our study, we could not fully neglect the potential heterogeneity. The subgroup analysis was used to assess the sources of heterogeneity. The results of subgroup analysis revealed that sample size, specimen type, source of HR and survival analysis mode might contribute to the heterogeneity. Therefore, more eligible studies are needed to explore the sources of heterogeneity. Moreover, the stability of our results was confirmed by sensitivity analysis.

In conclusion, the survival of MPM is significantly correlated with tumor histology and tumor stage. Furthermore, high soluble mesothelin level may lead to a poor prognosis for MPM patients. Therefore, it is appropriate to regard soluble mesothelin as an independent prognostic marker and a novel potential therapeutic target for MPM patients.

## MATERIALS AND METHODS

This meta-analysis was performed according to the statement of Preferred Reporting Items for Systematic Reviews and Meta-Analyses (PRISMA) [39].

### Search strategy

Two investigators independently searched the Medline (via OvidSP), Embase(via OvidSP), COCENTRAL and Pubmed for studies that investigated the prognosis with soluble mesothelin in MPM. Studies were examined, and an updated search was conducted on November 2016. The search terms were as follows: mesothelin and mesothelioma. The title and abstract of each identified study were browsed to exclude any irrelevant publications. The full texts of all potentially eligible studies were retrieved, and their references were carefully browsed to find other studies that met the criteria. Disagreements between two investigators were settled by discussion.

### Inclusion and exclusion criteria

Studies eligible for inclusion in this meta-analysis met the following criteria: (1) evaluated the prognostic value of soluble mesothelin, either in blood or pleural effusion(PE); (2) soluble mesothelin level was divided into high and low grades; (3) the literature should be published with full texts. Exclusion criteria included: (1) articles published as reviews, conference abstracts or comments;(2) studies with insufficient survival data for which HR and CI could not be determined;

### Data extraction and quality assessment

Two investigators extracted useful information from eligible studies independently. The following information was recorded: first author surname, publication year, country, study type, number of patients, ethnicity, tumor site, tumor histology, tumor stage, treatment, specimen, method, cut-off value, source of HR (95% CI), survival analysis mode. Hazard ratio was the most appropriate statistic to analyze time-to-event outcomes, because both the number of events and time of events were taken into account. When the HR with 95% was available from the original article, we used the data directly. Otherwise we extracted data from the Kaplan-Meier survival curves by the software Engauge Digitizer Version 4.1 (http://digitizer.sourceforge.net/). The method proposed by Tierney et al. was used to calculated the HR and 95%CI [40]. When both univariate and multivariate analysis were conducted in the studies, we preferred the results based on multivariate analysis. Disagreements were settled by discussion. The Newcastle-Ottawa Scale (NOS) was used to evaluate the quality of each individual study [41]. The NOS consists of three parameters of quality: selection (there terms: representativeness of the exposed cohort, selection of the non exposed cohort, selection of the non exposed cohort, demonstration that outcome of interest was not present at start of study?), comparability (one term: comparability of cohorts on the basis of the design or analysis), and outcome assessment (three terms: assessment of outcome, was follow-up long enough for outcomes to occur? adequacy of follow up of cohorts). The NOS score was ranged from 0 to 9, and the study with an NOS score ≥ 6 was considered as a high quality study.

### Statistical analysis

Hazard ratio (HR) with 95% CI was performed to estimate the prognostic value of soluble mesothelin and the effect of clinicopathological characteristics on the survival of MPM patients. Cochrane's Q test (Chi-squared test) and I^2^ metric were performed to calculate the statistical heterogeneity of the pooled HR with 95% CI [42]. For I^2^ statistics, I^2^<25% indicates there is a low heterogeneity, I^2^= 25%-50% indicates there is a moderate heterogeneity and I^2^ >50% indicates there is a significant heterogeneity [43]. The fixed-effect model was adopted in the meta-analysis when no statistically significant heterogeneity was observed between studies (p≥0.1 or I^2^ <50%), otherwise the random-effect model was used. When HR >1, a poor prognosis for MPM patients was indicated by high soluble mesothelin level. If there was no overlap between 95% CI and 1, it would be considered that the impact of soluble mesothein on survival was statistically significant. Subgroup analysis was stratified by specimen type, cut-off value, sample size, source of HR and survival analysis mode. The sensitivity analysis was performed using the leave-one-out method to explore the influence of each individual study on the pooled HR for overall survival. Publication bias was evaluated by funnel plot(qualitative) and Begg's test(quantitative). When publication bias existed, the nonparametric trim and fill method was applied to re-estimate a corrected effect size after adjustment for publication bias [44]. All p values were based on two-sided test, p value < 0.05 was considered to be statistically significant. STATA 11.0 software (Stata Corporation, College Station, TX) was used to conduct all statistical analysis.

## Abbreviations

MPM: malignant pleural mesothelioma
CI: confidence interval
HR: hazard ratio
